# Beyond the surface: a color-inclusive guide to central line site assessment

**DOI:** 10.1017/ash.2024.39

**Published:** 2024-04-03

**Authors:** Meredith Herrera, Siobhan Eichenblat, Eileen Campbell, Julia Shick, Heather Brown, Chelsea Brinkley, Shelley Kester, Jessica Layell, Catherine L Passaretti, Mindy M Sampson

**Affiliations:** 1 Department of Infection Prevention, Atrium Health, Charlotte, NC, USA; 2 Department of Nursing Professional Development, Atrium Health, Charlotte, NC, USA; 3 Section on Infectious Diseases, Department of Internal Medicine, Wake Forest University School of Medicine, Winston-Salem, NC, USA; 4 Division of Infectious Diseases & Geographic Medicine, Department of Medicine, Stanford University, Palo Alto, CA, USA

## Abstract

Significant gaps exist in representation of diverse populations in central-line assessment education and tools. We review some of these gaps and provide some real-world guidance on how to assess central line sites in patients of all skin tones.

Patients who develop a central line-associated bloodstream infection (CLABSI) or hospital-onset bacteremia (HOB) incur higher medical costs, longer lengths of stay, and increased mortality.^1,2^ While more studies are needed, existing evidence suggests there are inequities in CLABSI and HOB, with higher rates in patients of color compared to White patients.^3^ Notably, these inequities persist even after adjustment for patient-specific comorbidities and risk factors.^4,5^ McGrath et al. showed improvement in CLABSI rates in Black patients after implementing quality improvement interventions that included a focus on increasing the proportion of central line (CL) maintenance audits in patients of color. Few other studies have evaluated how to mitigate identified inequities.^6^

Over the last few decades, hospitals have made great improvements in preventing CLABSI.^7^ Standardizing techniques in insertion and maintenance of central lines have been key contributors to longitudinal improvement.^8,9^ Providing initial and ongoing education on CL maintenance to staff who care for these devices has long been recommended by the Centers for Disease Control and Prevention and remains in the 2022 CLABSI prevention recommendations.^10,11^ While the importance of staff education and competency in assessing and maintaining the CL site has been emphasized, current resources commonly focus on findings of infection or skin inflammation in patients with lightly pigmented skin. In parallel with the insufficient inclusion of patients with diverse skin tones in images across dermatology and nursing literature,^12–15^ infection prevention education frequently neglects to include varying ways infected or inflamed skin can present in patients with darkly pigmented skin.

To assess the current state of tools available for evaluation of CL sites in patients of all skin colors, we convened a multidisciplinary team including infection preventionists, a dermatology physician, infectious disease physicians, and nursing representatives. A literature search was performed to identify evidence-based resources that address the assessment of central lines in patients of all skin colors. While general dermatology publications on the recognition of cutaneous infection and inflammation in patients with darkly pigmented skin were identified, literature on CL assessment that specifically addressed issues particular to patients with darkly pigmented skin was lacking. Similarly, pictures and models used in publicly available educational tools predominantly included patients with White or lightly pigmented skin.^16–26^ Outreach to representatives from professional groups such as the Association for Professionals in Infection Control and Epidemiology and central line dressing vendors did not yield any additional relevant tools or resources.

Given the paucity of premade resources, representatives from nursing and infection prevention intentionally focused on assessing patients with a variety of skin tones during CL maintenance audits to improve assessment techniques. Through literature review, collaborative discussion, information accumulated through expert engagement, and CL site image review, tips for assessing CLs in patients of color were developed (Figure 1).

**Figure 1.**
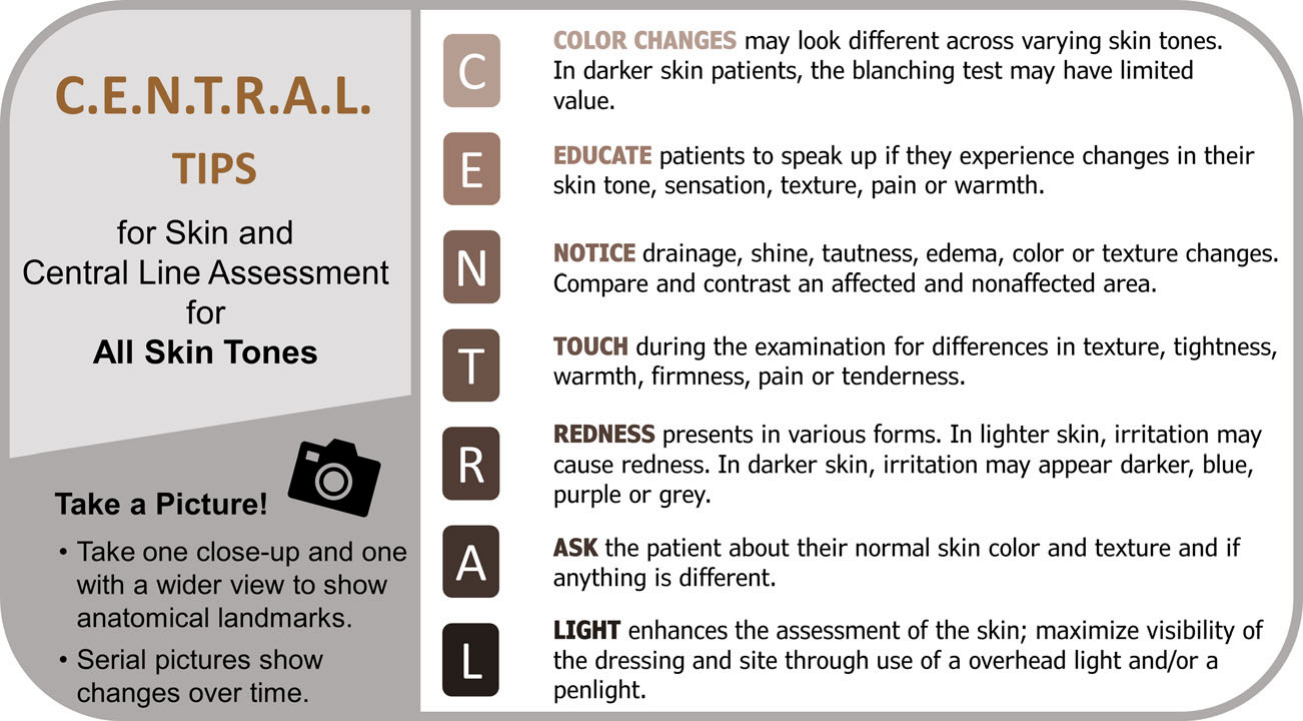
Tips for assessing central lines in varying skin tones.

## Tips for central line assessment for all skin tones

A commonly acknowledged early indication of infection at the site of CL insertion is the presence of erythema.^17^ While erythema may be readily apparent in patients with lightly pigmented skin, recognition of signs of infection may be more challenging in patients with darkly pigmented skin.^27^ Physician inter-rater reliability on skin assessment is poor for patients with higher levels of skin pigmentation.^28^ Skin changes at sites of infection result from heightened inflammation and increased blood flow in the area.^29^ In individuals with darkly pigmented skin, inflammation can alter the skin to appear more purple, brown, black, or gray.^27,30^ This color change produces less contrast between the affected and unaffected skin, making detection of early signs of infection challenging. This can lead to missed or delayed diagnosis. Assessment beyond visual inspection is critical to recognize inflammation and prevent CLABSI. Healthcare professionals must be aware of other cardinal signs of inflammation such as warmth, swelling, and pain, in addition to the variety of ways infection can present in all skin tones.^31^

1. Visual examination:
  1. Examine and compare: Increased shine, tautness of the skin, and color change (redness, bluish-purple discoloration, gray tone, or hyperpigmentation) can be signs of inflammation and infection. Comparison of the skin around the line to nonimpacted skin can help identify subtle differences.
  2. Lighting matters: Ensure the room lights are on and where possible use an additional source of light to optimally see the site you are evaluating. Fluorescent lights may give skin a blue tint, so using bright light or a penlight is ideal to see the true skin color most accurately.
  3. Pictures: Serial pictures of line sites over time can be helpful in detecting changes. Take one picture close-up to capture details and one picture with a wider view to include anatomic landmarks.
2. Palpation:
  1. Assess changes in texture, firmness, swelling, warmth, induration, and tenderness through physical touch and compare them to characteristics of unaffected skin.
  2. Be aware that the traditional “blanch test” may not be positive in individuals with darkly pigmented skin.^32^ This test assesses blood flow by applying pressure and evaluating if the area turns white or pale for longer than normal.
3. Gathering patient input:
  1. Whenever possible, ask what is normal for the patient and if they have noticed any skin changes, pain, or tenderness at the line site.
  2. Be cognizant of cultural or social customs that can cause changes to skin.
  3. Ensure patient communication is culturally appropriate and consider any educational or language barriers that may impact comprehension.

## Next steps and a call to action

The development and implementation of education is one step on the journey of bringing to light unintentional differences in patient care and, subsequently, achieving equity in care. Patient feedback and engagement on educational content and further opportunities to improve care for all patients, while often overlooked, is a key next step in this work. In addition, skin color is only one dimension of the patient that may impact differential quality in central line assessment. Other factors such as obesity and language, educational or cultural barriers between patients, and healthcare workers necessitate future exploration.

As with any educational effort in healthcare, adult learning principles and the variety of learning styles must be considered when developing educational methods to address the knowledge, skill, or practice gap. While distribution and communication of content in printed materials is important, it may not be effective for sustained change. Diverse skin assessment content, such as images of CL sites in skin of color, should be incorporated into orientation, annual regulatory competencies, and, perhaps most importantly, microlearning environments such as bedside rounding. Interactive case-based scenarios in which healthcare professionals are guided through the accurate evaluation of CL in patients with a variety of skin pigmentations may be particularly effective in improving clinical skills.

Where possible, sharing data stratified by sociodemographic factors for CLABSI outcomes and CL maintenance-related process measures can improve awareness and reinforce inclusive educational efforts. Standardized CL assessments with a focus on patients of various skin tones and feedback to staff with real-world pictures can help solidify concepts. Finally, many of the CL educational tools that are publicly available do not reflect a diverse patient population, and literature within healthcare epidemiology on healthcare disparities is limited. Moving consciously towards inclusion in all aspects of infection prevention and antibiotic stewardship is necessary to make sure we are providing equitable healthcare to all.

## References

[1] Stevens V , Geiger K , Concannon C , Nelson RE , Brown J , Dumyati G. Inpatient costs, mortality and 30-day re-admission in patients with central-line-associated bloodstream infections. Clin Microbiol Infect 2014;20:O318–324. 24112305 10.1111/1469-0691.12407

[2] Yu KC , Jung M , Ai C. Characteristics, costs, and outcomes associated with central-line-associated bloodstream infection and hospital-onset bacteremia and fungemia in US hospitals. Infect Control Hosp Epidemiol 2023;44:1920–1926. 37424226 10.1017/ice.2023.132 PMC10755163

[3] Willer BL , Tobias JD , Suttle ML , et al. Trends of racial/ethnic disparities in pediatric central line-associated bloodstream infections. Pediatrics 2022;150:e2021054955. 35979730 10.1542/peds.2021-054955

[4] McGrath CL , Bettinger B , Stimpson M , et al. Identifying and mitigating disparities in central line-associated bloodstream infections in minoritized racial, ethnic, and language groups. *JAMA Pediatr* 2023;177:700–709. 10.1001/jamapediatrics.2023.1379 PMC10230370 37252746

[5] Rha B , See I , Dunham L , et al. Vital signs: health disparities in hemodialysis-associated staphylococcus aureus bloodstream infections - United States, 2017-2020. MMWR Morb Mortal Wkly Rep 2023;72:153–159. 36757874 10.15585/mmwr.mm7206e1 PMC9925139

[6] Chen J , Khazanchi R , Bearman G , et al. Racial/Ethnic inequities in healthcare-associated infections under the shadow of structural racism: narrative review and call to action. Curr Infect Dis Rep 2021;23:17. 34466126 10.1007/s11908-021-00758-x PMC8390539

[7] Pronovost PJ , Cleeman JI , Wright D , et al. Fifteen years after To Err is Human: a success story to learn from. BMJ Qual Saf 2016;25:396–399. 10.1136/bmjqs-2015-004720 PMC6487657 26669931

[8] Wei AE , Markert RJ , Connelly C , et al. Reduction of central line-associated bloodstream infections in a large acute care hospital in Midwest United States following implementation of a comprehensive central line insertion and maintenance bundle. J Infect Prev 2021;22:186–193. 34659456 10.1177/17571774211012471 PMC8512874

[9] Redstone CS , Zadeh M , Wilson MA , et al. A quality improvement initiative to decrease central line-associated bloodstream infections during the COVID-19 pandemic: a “Zero Harm” approach. J Patient Saf 2023;19:173–179. 36849451 10.1097/PTS.0000000000001107 PMC10044591

[10] Buetti N , Marschall J , Drees M , et al. Strategies to prevent central line-associated bloodstream infections in acute-care hospitals: 2022 Update. Infect Control Hosp Epidemiol 2022;43:553–569. 35437133 10.1017/ice.2022.87 PMC9096710

[11] O’Grady NP , Alexander M , Burns LA , et al. Guidelines for the prevention of intravascular catheter-related infections. Am J Infect Control 2011;39:S1–34. 21511081 10.1016/j.ajic.2011.01.003

[12] Bellicoso E , Quick SO , Ayoo KO , Beach RA , Joseph M , Dahlke E. Diversity in dermatology? An assessment of undergraduate medical education. J Cutan Med Surg 2021;25:409–417. 33849302 10.1177/12034754211007430

[13] Pusey-Reid E , Quinn LW , Wong J , et al. Representation of dark skin tones in foundational nursing textbooks: an image analysis. Nurse Educ Today 2023;130:105927. 37556863 10.1016/j.nedt.2023.105927

[14] Lester JC , Taylor SC , Chren MM. Under-representation of skin of colour in dermatology images: not just an educational issue. Br J Dermatol 2019;180:1521–1522. 31157429 10.1111/bjd.17608

[15] Chatrath S , Bradley L , Kentosh J. Dermatologic conditions in skin of color compared to white patients: similarities, differences, and special considerations. Arch Dermatol Res 2023;315:1089–1097. 36450934 10.1007/s00403-022-02493-2

[16] New South Wales Government. Central line insertion online training. https://www.cec.health.nsw.gov.au/keep-patients-safe/infection-prevention-and-control/resources/cli-online-training. Published 2020. Accessed February 12, 2024.

[17] Gohil SK , Yim J , Quan K , et al. Impact of a Central-Line Insertion Site Assessment (CLISA) score on localized insertion site infection to prevent Central-Line-Associated Bloodstream Infection (CLABSI). Infect Control Hosp Epidemiol 2020;41:59–66. 31699181 10.1017/ice.2019.291

[18] MEDLINE. Central line bundle use: share this checklist with your team. https://www.medline.com/strategies/infection-prevention/central-line-bundle-implementation-checklist/. Published 2023. Accessed February 14, 2024.

[19] Dumyati G. Central line maintenance. https://www.urmc.rochester.edu/medialibraries/urmcmedia/community-health/research/communicable-disease-surveillance/healthcare-associated-infections/documents/clabsipresentationdumyati.pdf. Published 2012. Accessed February 14, 2024.

[20] Jones K. Maintenance and removal of central venous catheters. https://www.cdc.gov/infectioncontrol/pdf/strive/CLABSI104-508.pdf. Last Reviewed 2023. Accessed February 14, 2024.

[21] 3M. Help prevent bloodstream infections before they start. https://www.3mcanada.ca/3M/en_CA/medical-ca/conditions/bloodstream-infections/. Published 2011. Accessed February 14, 2024.

[22] Broadhurst D , Moureau N , Ullman AJ , et al. Management of central venous access device-associated skin impairment: an evidence-based algorithm. J Wound Ostomy Continence Nurs 2017;44:211–220. 28353488 10.1097/WON.0000000000000322 PMC5417573

[23] Vascular Wellness. PICC line and midline catheter care. https://www.vascularwellness.com/picc-line-and-midline-catheter-care/. Published 2021. Accessed February 14, 2024.

[24] Cincinnati Children’s. Central line care. https://www.cincinnatichildrens.org/health/c/central-line-care. Published 2022. Accessed February 14, 2024

[25] AmericanNurse. Color awareness: a must for patient Assessment. https://www.myamericannurse.com/color-awareness-a-must-for-patient-assessment/. Published 2011. Accessed February 14, 2024.

[26] Science Museum Group. Nightingale central venous catheter care instructions. https://collection.sciencemuseumgroup.org.uk/objects/co8820910/nightingale-central-venous-catheter-care-instructions-sign. Published 2020. Accessed February 14, 2024.

[27] Pusey-Reid E , Quinn L , Samost ME , et al. Skin assessment in patients with dark skin tone. Am J Nurs 2023;123:36–43. 10.1097/01.NAJ.0000921800.61980.7e 36815818

[28] Zhao CY , Wijayanti A , Doria MC , et al. The reliability and validity of outcome measures for atopic dermatitis in patients with pigmented skin: a grey area. Int J Womens Dermatol 2015;1:150–154. 28491979 10.1016/j.ijwd.2015.05.002 PMC5418878

[29] Koeppen B. Properties of the vasculature. In: Koeppen BN , Stanton BA , eds. *Berne & Levy Physiology*, Eighth Edition. Philadelphia, PA: Elsevier; 2023:342–382.

[30] Zhao CY , Hao EY , Oh DD , et al. A comparison study of clinician-rated atopic dermatitis outcome measures for intermediate- to dark-skinned patients. Br J Dermatol 2017;176:985–992. 28012183 10.1111/bjd.15271

[31] Wong CM , Scheufele CJ , Bodapativ S , et al. Presentations of cutaneous disease in various skin pigmentations: cutaneous abscesses. HCA Healthc J Med 2022;3:153–159. 37424603 10.36518/2689-0216.1431 PMC10324847

[32] Matas A , Sowa MG , Taylor V , Taylor G , Schattka BJ , Mantsch HH. Eliminating the issue of skin color in assessment of the blanch response. Adv Skin Wound Care 2001;14:180–188. 11902343 10.1097/00129334-200107000-00010

